# Plasma sICAM-1 as a Biomarker of Carotid Plaque Inflammation in Patients with a Recent Ischemic Stroke

**DOI:** 10.1007/s12975-022-01002-x

**Published:** 2022-03-02

**Authors:** Núria Puig, Pol Camps-Renom, Mercedes Camacho, Ana Aguilera-Simón, Francesc Jiménez-Altayó, Alejandro Fernández-León, Rebeca Marín, Joan Martí-Fàbregas, Jose Luis Sánchez-Quesada, Elena Jiménez-Xarrié, Sonia Benitez

**Affiliations:** 1 Cardiovascular Biochemistry, Biomedical Research Institute Sant Pau (IIB-Sant Pau), Barcelona, Spain; 2 Department of Biochemistry and Molecular Biology, Faculty of Medicine, Building M, Universitat Autònoma de Barcelona (UAB), Cerdanyola del Vallès, Barcelona, Spain; 3 Stroke Unit, Department of Neurology, Hospital de La Santa Creu I Sant Pau, IIB-Sant Pau, Barcelona, Spain; 4 Genetic of Complexes Diseases, Biomedical Research Institute Sant Pau (IIB-Sant Pau), Barcelona, Spain; 5 Department of Pharmacology, Neuroscience Institute, Faculty of Medicine, UAB, Cerdanyola del Vallès, Barcelona, Spain; 6 Department of Nuclear Medicine, Hospital de La Santa Creu I Sant Pau, IIB-Sant Pau, Barcelona, Spain; 7 CIBER of Diabetes and Metabolic Diseases (CIBERDEM), Madrid, Spain

**Keywords:** Ischemic stroke, ^18^F-FDG PET, Inflammatory biomarkers, sICAM-1, Carotid plaque

## Abstract

^18^F-fluorodeoxyglucose positron emission tomography (^18^F-FDG PET) identifies carotid plaque inflammation and predicts stroke recurrence in patients with atherothrombotic stroke. The aim of the study was to identify plasma inflammatory biomarkers associated with plaque inflammation according to ^18^F-FDG uptake. We conducted a prospective study of consecutive adult patients with a recent (< 7 days) anterior circulation ischemic stroke and at least one atherosclerotic plaque in the ipsilateral internal carotid artery. We included 64 patients, 57.8% of whom showed a carotid stenosis ≥ 50%. All patients underwent an early (< 15 days from inclusion) ^18^F-FDG PET, and a blood sample was obtained at days 7 ± 1 from the stroke. The plasma concentration of 16 inflammation-related molecules was analyzed in a Luminex using xMAP technology. Multivariable linear regression was used to assess the association between plasma biomarkers and the standardized uptake value (SUV) of ^18^F-FDG uptake. Soluble intercellular adhesion molecule-1 (sICAM-1), soluble vascular adhesion molecule-1 (sVCAM-1), and fractalkine (FKN) were independently associated with plaque inflammation (*β* = 0.121, 95% *CI* 0.061–0.181, *p* < 0.001; *β* = 0.144, 95% *CI* 0.012–0.276, *p* = 0.033; *β* = 0.136, 95% *CI* 0.037–0.235, *p* = 0.008). In a multivariable logistic regression analysis, sICAM-1 was associated with SUVmax ≥ 2.85 (*OR* = 1.02, 95% *CI* 1.00–1.03, *p* = 0.020). Multivariable Cox regression was used to assess the association between biomarkers and stroke recurrence. sICAM-1 was associated with stroke recurrence (*HR* = 1.03, 95% *CI* 1.00–1.05, *p* = 0.002). In summary, elevated concentrations of sICAM-1 were associated with carotid plaque inflammation and an increased risk of stroke recurrence in patients with recent ischemic stroke and carotid atherosclerosis.

**Supplementary Information:**

The online version contains supplementary material available at 10.1007/s12975-022-01002-x.

## Introduction

Ischemic stroke is a leading cause of death and disability worldwide. Approximately, 20% of all ischemic strokes are attributed to atherosclerosis, being the plaques from the internal carotid artery the most frequently involved. Symptomatic carotid plaques are associated with a threefold risk of early stroke recurrence compared to the risk of recurrence in other stroke subtypes; therefore, secondary stroke prevention after an initial atherothrombotic stroke is of huge importance.

Besides intensive medical management, secondary prevention strategies after an ischemic stroke related to carotid atherosclerosis include surgical revascularization procedures, mainly carotid stenting or carotid endarterectomy. Currently, the decision to apply an invasive carotid revascularization procedure mostly depends on the degree of carotid stenosis [1, 2]. However, the degree of stenosis alone is insufficient for deciding upon the best treatment in some common clinical situations, such as patients with vulnerable plaques causing less than 50% of stenosis [3] or female patients with moderate carotid stenosis (50–69%) [4, 5]. Therefore, there is a need for complementary tools that can help to stratify patients at high risk of stroke recurrence and those who might benefit from carotid revascularization. Accordingly, several investigations have been conducted in the past years to validate new imaging and plasma biomarkers to detect carotid plaque vulnerability. Most of them have focused on inflammation as a growing body of evidence shows that inflammation is a key factor for atherosclerosis and plaque vulnerability.

Carotid ^18^F-fluorodeoxyglucose positron emission tomography (^18^F-FDG PET) has emerged as one of the most promising new imaging biomarkers of vascular inflammation. ^18^F-FDG PET allows for the quantification of carotid plaque inflammation by detecting macrophage activation with an excellent histological correlation [6]. An independent association between plaque ^18^F-FDG uptake and early stroke recurrence in patients with carotid stenosis was reported in a large multicenter prospective cohort study [7]. In that study, there was a twofold increase in the risk of stroke recurrence per 1 g/mL in ^18^F-FDG uptake at the site of maximum inflammation within the plaque. A maximal standardized uptake value (SUVmax) threshold of 2.85 g/mL gave the optimal balance of sensitivity and specificity for discrimination of stroke recurrence, even in patients with moderate stenosis. A risk score including SUVmax and carotid stenosis improved the identification of patients at a high risk of stroke recurrence [8]. These studies were the first proofs of concept showing that an assessment of carotid plaque inflammation adds valuable information to the decision-making approach for stroke patients. However, the high cost and limited availability of ^18^F-FDG PET may hinder the widespread implementation of this technique in clinical practice.

In addition to imaging techniques, some inflammatory plasma biomarkers have been studied as plausible markers of carotid plaque vulnerability. Inflammatory molecules are related to the development and progression of atherosclerotic plaque, and macrophages play a key role in the release of these inflammatory mediators, including first-wave inflammatory cytokines: interleukins-1β and -6 (IL-1β, IL-6) and tumor necrosis factor-α (TNF-α); chemokines, which are involved in inflammatory cell attraction: IL-8, monocyte chemoattractant protein-1 (MCP-1), and fractalkine (FKN); soluble adhesion molecules: soluble intercellular adhesion molecule 1 (sICAM-1), soluble vascular adhesion molecule 1 (sVCAM-1), and soluble P-selectin (sP-selectin); and matrix metalloproteinases (MMP) involved in extracellular matrix degradation: MMP1, MMP2, MMP8, and MMP9 [9].

There is controversy regarding the value of blood inflammatory markers in predicting initial or recurrent strokes in clinical settings. Some clinical studies have reported independent associations of inflammatory markers, such as C-reactive protein (CRP), and vascular events; however, conflicting results have been found regarding recurrent strokes [10, 11]. In the CANTOS trial, the IL-1β antagonist canakinumab reduced recurrent vascular events in patients with stable coronary disease; however, no significant results were found in patients with stroke [12]. Thus, further investigations are still needed on the role of inflammatory biomarkers in stroke patients with carotid atherosclerosis.

In the present study, we hypothesized that some inflammatory plasma biomarkers in patients with a recent ischemic stroke and carotid atherosclerosis are associated with carotid plaque inflammation assessed by ^18^F-FDG PET. In this context, we aimed to determine the following: (1) which plasma biomarkers within a portfolio of inflammatory molecules were specifically elevated in stroke patients with carotid atherosclerosis compared to healthy controls, (2) which of those molecules were associated with ^18^F-FDG PET, and (3) whether some of those molecules might also predict stroke recurrence. To the best of our knowledge, no previous studies have addressed the last two points, which are essential and deserve to be investigated.

## Methods

### Study Design

We conducted an observational cohort study (NCT03218527) of consecutive adult patients with a recent anterior circulation ischemic stroke and carotid atherosclerosis and a control group comprised of healthy subjects. The study was conducted in our center between January 2016 and March 2019. It was approved by the Ethics Committee of Hospital Santa Creu i Sant Pau, and the patients gave written consent to participate. The study was performed in accordance with the Helsinki Declaration.

### Study Population

Patients were included in the study if they fulfilled the following criteria: (1) age ≥ 50 years in order to minimize the inclusion of non-atherosclerotic vasculopathies [7, 8]; (2) anterior circulation ischemic stroke or transient ischemic attack (TIA) within 7 days before inclusion; and (3) at least one atherosclerotic plaque in the internal carotid artery (ICA) on the side consistent with stroke symptoms, regardless of the degree of stenosis. Carotid stenosis was graded using the NASCET approach [13] with computed tomography (CT) or magnetic resonance imaging angiography when available, as well as based on hemodynamic criteria using ultrasound [14]; and (4) modified Rankin Scale (mRS) score < 4 before inclusion. Exclusion criteria were as follows: (1) the presence of definitive cardioembolic, lacunar, or unusual stroke etiology according to the TOAST criteria [15], (2) the presence of a hemodynamic stroke/TIA, (3) prior carotid surgery or stenting, (4) the presence of comorbidities conditioning a life-expectancy < 1 year, and (5) suspicion of concomitant infections at the time of blood extraction.

The following variables were recorded for all of the patients:

1. Age and sex
2. Past medical history including hypertension, diabetes, dyslipidemia, prior stroke/TIA, coronary artery disease, tobacco, and alcohol consumption
3. Previous treatments
4. National Institutes of Health Stroke Scale (NIHSS) score, as a surrogate of infarct size
5. Body mass index (BMI), which was divided into three categories (healthy weight, BMI 18.5 to < 25; overweight, BMI 25 to < 30; and obesity, BMI ≥ 30)
6. Regular physical exercise according to the physician-based assessment and counseling for exercise (PACE) scale [16]
7. Mediterranean diet adherence according to the PREDIMED score [17]
8. mRS score at inclusion
9. Stroke etiology according to the TOAST criteria [15] after a diagnostic workup that included at least a 24-h electrocardiogram, an echocardiogram, and an ultrasound carotid examination (assessing plaque echolucency, plaque surface, and degree of stenosis by hemodynamic criteria)
10. Results from the admission blood test including renal function, hemogram, hemostasis, and lipid profile

Healthy controls were included in the control group if they fulfilled the following criteria: (1) age ≥ 50 years, (2) no prior history of ischemic heart disease, and (3) no prior history of stroke. All the control participants underwent a clinical interview to assess demographics, lifestyle habits, and prior treatments. Additionally, a carotid ultrasound examination was performed to rule out the presence of asymptomatic high degree stenosis in both ICA and an electrocardiogram to rule out silent ischemic heart disease and atrial fibrillation. Healthy subjects did not undergo ^18^F-FDG PET examination.

### Procedures

All stroke patients underwent a baseline medical assessment by a trained study personnel, ^18^F-FDG PET/CT < 15 days from the index stroke, and blood sample collection at 7 ± 1 days from the index stroke. The treating clinicians were advised to provide medical and revascularization treatments according to the guidelines [18].

### ^18^F-FDG PET Imaging and Assessment of Plaque Inflammation

Carotid ^18^F-FDG PET was performed in a Philips Gemini TF TOF 64 PET/CT (Philips Medical System, Eindhoven, Netherland). The examinations were performed after a fast that lasted a minimum of 6 h. PET scans were not performed if pre-PET blood glucose exceeded 10 mmol/L. Two hours before image acquisition, 320 MBq of ^18^F-FDG was administered. The uptake phase was standardized with the patient resting. PET images were acquired in 3-dimensional mode in 2-bed positions for 10 min each. CT angiography was done at admission, usually some days before PET/CT. Images from CT angiography and PET were co-registered afterwards to assess the slice of maximal plaque stenosis.

^18^F-FDG activity was measured in 10 regions of interest, which were defined relative to the slice of maximal stenosis on the co-registered CT angiography, corresponding to a 1-mm axial plaque slice. ^18^F-FDG was quantified using standardized uptake values (SUV g/mL, defined as measured uptake [MBq/mL]/injected dose [MBq] per patient weight [g]). We defined the single hottest slice as the axial slice with maximal SUV uptake (SUVmax).

### Plasma and Serum Determinations

Peripheral blood samples from the stroke patient were collected at days 7 ± 1 from the stroke/TIA. Plasma was collected in ethylenediaminetetraacetic acid (EDTA)-containing Vacutainers, and serum was collected in serum separator tubes coated with clot activator. Tubes were centrifuged at 1500 g for 15 min at 4 °C, and aliquots were frozen at − 80 °C until analysis. The same blood collecting protocol was used for the healthy controls.

Serum lipid profile (triglycerides, total cholesterol, low-density lipoprotein cholesterol (LDLc) and high-density lipoprotein cholesterol (HDLc)), creatinine, hemoglobin, and high sensitivity CRP (hsCRP) were measured in an autoanalyzer (Alinity ci-series, Abbott Core Laboratory, Chicago, Illinois, USA). Glycated hemoglobin (Hba1c) was quantified using high-performance liquid chromatography in total blood.

The plasma concentrations of fatty acid-binding protein 4 (FABP4), sP-selectin, sICAM-1, sVCAM-1, MMP1, MMP2, MMP9, FKN, granulocyte–macrophage colony-stimulating factor (GM-CSF), IL-10, IL-1β, IL-6, IL-8, macrophage inflammatory protein-3 alpha (MIP3A), and TNF-α were analyzed in a Luminex using xMAP® technology with a MILLIPLEX® MAP multiplexed assay kit (Merck Millipore, Burlington, MA, USA). MCP-1 was measured with a commercial ELISA kit (Invitrogen, Carlsbad, CA, USA).

### Outcomes and Follow-up

The primary outcome was inflammation assessed by ^18^F-FDG uptake in carotid PET examinations. The secondary outcome was ipsilateral recurrence stroke during the follow-up period. Patients were followed up at 3 months and at 1 year after the index stroke. A recurrent stroke was defined as a new sudden onset of persistent (> 24 h) neurological deficit after the index event or a new persistent or transient neurological deficit with imaging confirmation of a new cerebral infarction.

### Statistical Analysis

Continuous descriptive variables were reported as means and standard deviations (SD) or medians (md) and interquartile ranges (IQR) if they were not normally distributed. Categorical variables were expressed as counts and percentages. We divided the stroke population into two groups: < 50% stenosis and ≥ 50% stenosis. Differences between the stroke groups and the control were assessed using analysis of variance (ANOVA) or the Kruskal–Wallis rank-sum test (when a nonparametric test was required) for continuous variables and the *χ*^2^ test for categorical variables. Comparison of plasma molecule levels between the stroke patients and the control group was performed using the Wilcoxon rank-sum test.

Bivariate linear regression analysis, after logarithmic transformation of the variables (plasma molecules), was used to determine molecules associated with carotid plaque ^18^F-FDG uptake (SUVmax), expressed as *β*-coefficient with 95% confidence interval (CI). Backward stepwise multivariable linear regression modeling was performed considering each biomarker individually and including independent clinical variables based on a *p* < 0.1 on bivariate analysis.

Bivariate logistic regression analysis was used to determine biomarkers associated with the probability of presenting SUVmax ≥ 2.85 g/mL. The results were expressed as odds ratio (OR) with 95% CI. Once again, backward stepwise multivariable linear regression modeling was performed considering each biomarker individually and including independent clinical variables based on a *p* < 0.1 on bivariate analysis. Receiver operating characteristic (ROC) analysis was used to determine the sensitivity and specificity of different cutoff points of candidate biomarkers associated with the risk of SUVmax ≥ 2.85 g/mL.

Bivariate Cox regression analysis was used to determine factors associated with stroke recurrence during the follow-up, which was expressed as hazard ratio (HR) with 95% CI. Patients were censored earlier in the analysis if they presented a stroke recurrence or if they underwent carotid revascularization. Backward stepwise multivariable Cox regression modeling was also performed using the same approach as previously described.

Statistical significance for all the analyses was set at *p* < 0.05 (two-sided). Analyses were performed using Stata v.15 (Texas, USA).

## Results

### Clinical Characteristics

The study population included 37 patients with at least one atherosclerotic plaque causing  ≥ 50% of stenosis, 27 patients with a carotid stenosis < 50%, and 27 healthy controls. The clinical characteristics of these groups are detailed in Table 1. The groups were balanced for demographics and lifestyle habits except for regular physical exercise, which was more frequent in the control group, and current smoking, which was more frequent in the stroke patients. As expected, vascular risk factors, such as hypertension or diabetes, were also more frequently observed in the stroke patients than in the control group. Similarly, the stroke patients were more frequently treated with antiplatelet agents and statins; consequently, they had lower concentrations of total cholesterol, LDLc, and HDLc. In addition, hsCRP was elevated in the  ≥ 50% stenosis group compared to the control group. There were no significant differences between the stroke groups (< 50% stenosis vs. ≥ 50% stenosis) for any variable except the TOAST classification, which consistently indicated a greater proportion of atherothrombotic strokes in the ≥ 50% stenosis group and a higher proportion of cryptogenic strokes in the < 50% group. In the ≥ 50% stenosis group, 15 patients (40.5%) underwent carotid revascularization after the stroke. Reasons for not performing carotid revascularization included old age or other comorbidities contraindicating surgery (*n* = 18) and decision of the patient (*n* = 4).

**Table 1:** Clinical characteristics and biochemical parameters of the patients

	< 50% stenosis group (*n* = 27)	≥ 50% stenosis group (*n* = 37)	Control group (*n* = 27)	*p*
Age, mean (SD)	73.6 (9.7)	75.9 (9.7)	73.8 (5.7)	0.473
Sex (female), *n* (%)	5 (18.5)	11 (29.7)	6 (22.2)	0.563
Obesity (BMI ≥ 30), *n* (%)	2 (7.4)	5 (13.5)	4 (14.8)	0.925
Regular exercise (PACE ≥ 4), *n* (%)	13 (48.2)	17 (46.0)	20 (74.1)	0.058
PREDIMED score, md (IQR)	9 (5–10)	9 (7–9)	8 (7–9)	0.245
Current Smoking, *n* (%)	4 (14.8)	10 (27.0)	0 (0.0)	**0.012**
Hypertension, *n* (%)	26 (96.3)	27 (73.0)	18 (66.7)	**0.020**
Diabetes, *n* (%)	9 (33.3)	18 (48.7)	5 (18.5)	**0.043**
Dyslipidemia, *n* (%)	20 (74.1)	23 (62.2)	7 (25.9)	**0.001**
Active or recent cancer (< 5 years), *n* (%)	2 (7.4)	3 (8.1)	2 (7.4)	0.992
Coronary artery disease, *n* (%)	7 (25.9)	10 (27.0)	-	0.922
Prior stroke, *n* (%)	5 (18.5)	6 (16.2)	-	0.809
Prior antiplatelet therapy, *n* (%)	15 (55.6)	22 (59.5)	5 (18.5)	** < 0.001**
Prior statin therapy, *n* (%)	15 (55.6)	20 (54.1)	6 (22.2)	** < 0.001**
Baseline NIHSS, md (IQR)	3 (1–6)	2 (0–3)	-	0.324
Intravenous fibrinolysis, *n* (%)	7 (25.9)	4 (10.8)	-	0.113
Acute lesion on neuroimaging, *n* (%)	20 (74.1)	26 (70.3)	-	0.738
Stroke etiology, *n* (%)				
Atherothrombotic	0 (0.0)	31 (83.8)	-	** < 0.001**
Lacunar	7 (25.9)	0 (0.0)		
Cryptogenic	20 (74.1)	0 (0.0)		
Undetermined (two causes)	0 (0.0)	6 (16.2)		
Biochemical parameters				
Creatinin (mg/dL), md (IQR)	0.94 (0.79–1.05)	0.94 (0.77–1.13)	-	0.796
Haemoglobin (g/L), md (IQR)	144 (121–154)	134 (123–143)	147 (138–158)	0.057
HbA1c (%), md (IQR)	6 (5.8–7)	6 (5.7–7.2)	5.9 (5.5–6.4)	0.342
Triglycerides (mg/dL), md (IQR)	105 (82–143)	97 (77–134)	103 (78–127)	0.694
Total cholesterol (mg/dL), md (IQR)	153 (115–177)	145 (124–189)	179 (154–227)	**0.002**
LDLc (mg/dL), md (IQR)	76 (56–111)	86 (58–121)	102 (84–141)	**0.012**
HDLc (mg/dL), md (IQR)	40 (30–51)	30 (31–50)	51 (44–61)	** < 0.001**
hsCRP (mg/L), md (IQR)	3.1 (1.6–5.8)	6.1 (2.3–20)	2.0 (1.2–5.2)	**0.003**

*BMI* (body mass index); *PACE* (physician-based assessment and counseling for exercise); *NHISS* (National Institutes of Health Stroke Scale); *HbA1c* (glycated hemoglobin); *LDLc* (low-density lipoprotein cholesterol); *HDLc* (high-density lipoprotein cholesterol); *hsCRP* (high-sensitivity C-reactive protein). Analysis of variance (ANOVA) or the Kruskal–Wallis rank-sum test (when a nonparametric test was required) for continuous variables, and the *χ*^2^ test for categorical variables were used; *p* < 0.05 indicates significant differences between groups

### Study of the Plasma Inflammatory Molecules

#### Plasma Inflammatory Molecule Concentrations

Supplementary Table 1 summarizes the inflammatory biomarker concentrations by study groups. Remarkably, all biomarkers were elevated in the stroke population except for MMP2 and FABP4, which did not show significant differences compared to the control population. Importantly, no significant differences were found between the stroke groups for any of the biomarkers.

### Primary Outcome

^18^F-FDG PET was performed in accordance with the study protocol in 53 out of 64 patients (83%; 24 in the < 50% stenosis group and 29 in the ≥ 50% stenosis group). In three cases, ^18^F-FDG PET was performed; however, the carotid inflammation was not assessable due to acquisition problems. ^18^F-FDG PET was not performed in four cases because the patients underwent carotid revascularization before the PET examination, while four other patients refused to undergo PET.

Mean plaque SUVmax was 2.58 g/mL (*SD* = 0.54) in the < 50% stenosis group and 2.83 g/mL (*SD* = 0.57) in the  ≥ 50% stenosis group (*p* = 0.098). Table 2 shows the bivariate linear regression analysis of the association between each inflammatory biomarker and carotid inflammation (SUVmax). Among all of the analyzed molecules, sICAM-1 (*p* = 0.001) and FKN (*p* = 0.004) each had a significant association with carotid plaque inflammation, while sVCAM-1 (*p* = 0.063) showed a strong trend toward significance. Similar results were observed when the concentrations of these three molecules were divided by tertiles (data not shown). These three candidate biomarkers were selected for further multivariable analyses. Results from the bivariate and multivariable linear regression analyses of predictors of carotid plaque inflammation, including the clinical variables and the three candidate biomarkers, are shown in Table 3. The multivariable analyses showed that plasma concentrations of sICAM-1, sVCAM-1, and FKN independently predicted carotid plaque inflammation. These associations remained significant after adjusting for NIHSS.

**Table 2:** Bivariate linear regression analyses of the association between each inflammatory biomarker and carotid inflammation (SUVmax)

	*ß*-coefficient	95% CI	*p*
sP-selectin (ng/mL)	− 0.156	− 0.117–0.086	0.760
sICAM-1 (ng/mL)	0.121	0.055–0.187	**0.001**
sVCAM-1 (ng/mL)	0.134	− 0.008–0.245	0.063
MMP1 (ng/mL)	0.034	− 0.024–0.091	0.246
MMP9 (ng/mL)	0.006	− 0.064–0.076	0.871
FKN (pg/mL)	0.154	0.051–0.258	**0.004**
GM-CSF (pg/mL)	0.059	− 0.034–0.152	0.210
IL-10 (pg/mL)	0.042	− 0.034–0.120	0.280
Il-1β (pg/mL)	0.051	− 0.032–0.135	0.223
IL-6 (pg/mL)	0.002	− 0.034–0.038	0.920
IL-8 (pg/mL)	0.023	− 0.041–0.086	0.476
MIP3A (pg/mL)	0.052	− 0.050–0.153	0.315
TNF-α (pg/mL)	0.038	− 0.086–0.162	0.541
MCP-1 (pg/mL)	− 0.033	− 0.120–0.053	0.440

*SUVmax* (maximal standardized uptake value); *sP-selectin* (soluble P-selectin); *sICAM-1* (soluble intercellular adhesion molecule-1); *sVCAM-1* (soluble vascular cell adhesion molecule-1); *MMP* (matrix metalloproteinases); *FKN* (fractalkine); *GM-CSF* (granulocyte–macrophage colony-stimulating factor); *IL* (interleukin); *MIP3A* (macrophage inflammatory protein-3); *TNF-α* (tumor necrosis factor-α); *MCP-1* (monocyte chemoattractant protein-1). Logarithmic transformation of the variables, except in SUVmax, was used in bivariate linear regression analysis; *n* = 53; *p* < 0.05 indicates significant differences between groups

**Table 3:** Bivariate and multivariable linear regression analyses of predictors of carotid plaque inflammation measured by ^18^F-FDG PET (SUVmax)

	*β*-coefficient	95% CI	*β* Standardized	*p*
*Bivariate analysis*				
Age	0.001	− 0.001–0.004	0.125	0.372
Sex (female)	0.036	− 0.017–0.090	0.188	0.179
BMI category*	0.049	0.015–0.082	0.380	**0.005**
PACE score	− 0.015	− 0.028 to − 0.002	− 0.312	**0.023**
PREDIMED score	− 0.005	− 0.016–0.006	− 0.133	0.344
Current smoking	− 0.034	− 0.082–0.014	− 0.196	0.160
Hypertension	0.042	− 0.028–0.112	0.168	0.230
Diabetes	0.025	− 0.024–0.073	0.142	0.310
Dyslipidemia	0.019	− 0.033–0.071	0.103	0.464
Active or recent cancer (< 5 years)	0.091	0.003–0.178	0.281	**0.042**
Coronary artery disease	− 0.030	− 0.084–0.024	− 0.153	0.274
Prior stroke	− 0.030	− 0.094–0.033	− 0.132	0.347
Prior antiplatelet therapy	− 0.020	− 0.069–0.028	− 0.117	0.402
Prior statin therapy	0.003	− 0.048–0.049	0.002	0.990
Carotid stenosis ≥ 50%	0.257	− 0.050–0.564	0.229	0.098
Triglycerides (mg/dL)	0.003	− 0.002–0.001	0.199	0.166
Total cholesterol (mg/dL)	0.000	− 0.000–0.001	0.100	0.485
LDLc (mg/dL)	0.000	− 0.001–0.001	0.033	0.825
HDLc (mg/dL)	0.001	− 0.001–0.002	0.105	0.481
*Multivariable analysis*				
*Model 1 (including sICAM-1) R*^*2*^ = *0.399*				
sICAM-1 (ng/mL)	0.121	0.061–0.181	0.476	** < 0.001**
Carotid stenosis ≥ 50%	0.045	0.003–0.088	0.252	**0.037**
BMI category (× 1 category increase)	0.044	0.013–0.075	0.336	**0.006**
*Model 2 (including sVCAM-1)* *R*^*2*^ = *0.218*				
sVCAM-1(ng/mL)	0.144	0.012–0.276	0.290	**0.033**
BMI category (× 1 category increase)	0.050	0.015–0.084	0.381	**0.006**
*Model 3 (including FKN)* *R*^*2*^ = *0.263*				
FKN (pg/mL)	0.136	0.037–0.235	0.353	**0.008**
BMI category (× 1 category increase)	0.042	0.009–0.075	0.324	**0.015**

*SUVmax* (maximal standardized uptake value); *BMI* (body mass index); *PACE* (physician-based assessment and counseling for exercise); *LDLc* (low-density lipoprotein cholesterol); *HDLc* (high-density lipoprotein cholesterol); *sICAM-1* (soluble intercellular adhesion molecule-1); *sVCAM-1* (soluble vascular cell adhesion molecule-1); *FKN* (fractalkine). Logarithmic transformation of the variables, if they were not normally distributed, was used in bivariate and multivariable linear regression analysis; backward stepwise multivariable linear regression modeling was performed individually for each biomarker; *n* = 53; *p* < 0.05 indicates significant differences between groups. *BMI was divided into three categories (healthy weight, BMI 18.5 to < 25; overweight, BMI 25 to < 30; and obesity, BMI ≥ 30)

**Fig. 1:**
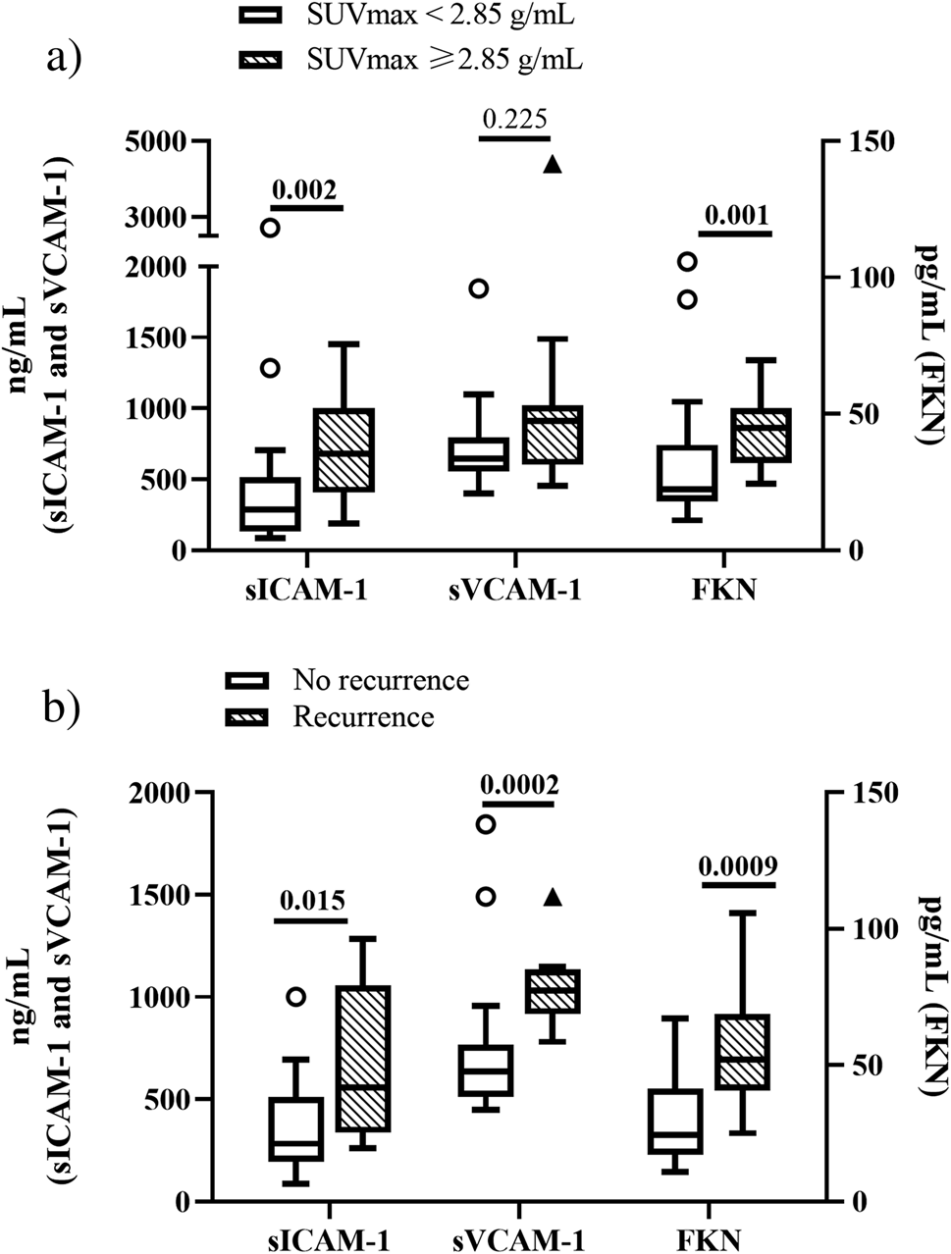
sICAM-1, sVCAM-1, and FKN concentrations in patients with a SUVmax < or ≥ 2.85 g/mL and in patients with no recurrence or with recurrence sICAM-1, sVCAM-1, and FKN concentrations in plasma from ischemic stroke patients with carotid atherosclerosis were measured with a MILLIPLEX® MAP multiplexed assay kit in Luminex. The values of concentration for each molecule were grouped according to those values from patients with a SUVmax < 2.85 g/mL (white boxes) and those from patients with a SUVmax ≥ 2.85 g/mL (striped boxes) (Fig. 1a). The values of concentration for each molecule were grouped according to those values from patients not suffering (white boxes) and suffering from recurrence (striped boxes). Data are shown as medians ± standard deviations; *n* = 38 for SUVmax < 2.85 g/mL and *n* = 15 for SUVmax ≥ 2.85 g/mL (A); *n* = 29 for non-stroke recurrence and *n* = 8 for stroke recurrence (Fig. 1b), *p*-value between groups is indicated in the figure

**Fig. 2:**
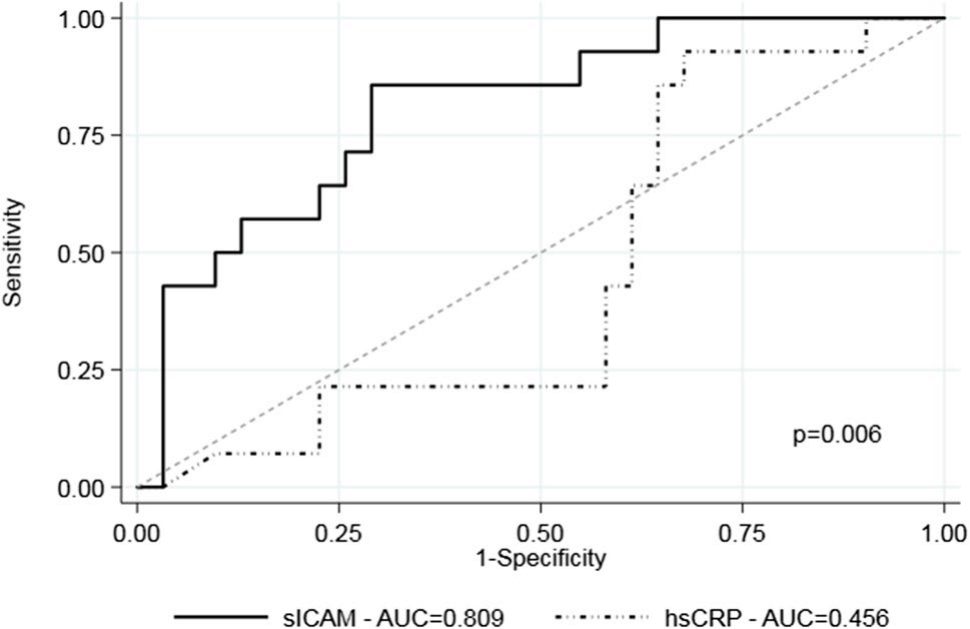
ROC analyses of sICAM-1 and CRP for discrimination of SUVmax ≥ 2.85 g/mL. Plasma sICAM-1 and CRP concentrations were measured in plasma from ischemic stroke patients with carotid atherosclerosis. sICAM-1 levels were measured with a MILLIPLEX® MAP multiplexed assay kit in Luminex, and CRP concentration was measured by hsCRP assay in an autoanalyzer. ROC curves for sICAM-1 (solid line) and hsCRP (dotted line) and their AUC are shown, *n* = 53

A high inflammation level, defined as a SUVmax ≥ 2.85 g/mL [7], was observed in 15 (28.3%) carotid plaques. Figure 1a shows that plasma concentrations of sICAM-1 and FKN were significantly higher in patients presenting a SUVmax ≥ 2.85 g/mL than in patients with a SUVmax < 2.85 g/mL. Supplementary Table 2 details the bivariate and multivariable logistic regression analyses of predictors of carotid plaque SUVmax ≥ 2.85 g/mL. Remarkably, in the final multivariable logistic regression model, plasma concentrations of sICAM-1 independently predicted the probability of finding a SUVmax ≥ 2.85 g/mL after adjusting for the PACE score, carotid stenosis, and BMI category. FKN association did not remain significant in the multivariable analysis. Figure 2 shows the ROC curve of sICAM-1 and hsCRP for discrimination of SUVmax ≥ 2.85 g/mL. The area under the curve (AUC) was 0.81 for sICAM-1 and 0.45 for hsCRP. A sICAM-1 threshold of 240 ng/mL had a sensitivity of 93.3% and a specificity of 42.4% for the discrimination of patients with a SUVmax ≥ 2.85 g/mL.

### Association of Inflammatory Molecules with Carotid Plaque Features

All the patients included in the study underwent also a baseline carotid ultrasound. Supplementary Table 3 summarizes the association between concentrations of the three candidate inflammatory biomarkers and plaque risk features assessed by ultrasonography. Remarkably, patients presenting predominantly hypoechoic plaques showed greater concentration of FKN compared to patients with hyperechoic plaques (median concentration 43 vs. 24 pg/mL; *p* = 0.025).

### Secondary Outcome

After a median follow-up of 10 (IQR 4–14) months, we registered stroke recurrences in 8 patients, all of whom were in the ≥ 50% stenosis group. In this group of patients, 15 patients were censored earlier because of carotid revascularization. Figure 1b shows that the plasma concentrations of the three candidate molecules in the ≥ 50% stenosis group (*n* = 37) were significantly higher in patients who suffered a recurrence than in those who did not. Table 4 shows the bivariate and multivariable Cox regression analyses of stroke recurrence predictors in patients with carotid stenosis ≥ 50%. In the multivariable Cox regression analysis, plasmatic concentrations of sICAM-1 were independently associated (*p* = 0.002) with stroke recurrence. This association remained significant after adjusting for age and NIHSS, but not after adjusting for SUVmax. Neither FKN nor sVCAM-1 predicted stroke recurrence in the multivariable Cox regression analysis.

**Table 4:** Bivariate and multivariable Cox regression analyses of predictors of stroke recurrence in patients with carotid stenosis ≥ 50%

*Bivariate analysis*			
	HR	95% CI	p
Age	0.99	0.92–1.07	0.786
Sex (female)	2.38	0.58.9.69	0.226
BMI category*	0.87	0.30–2.53	0.794
PACE score	0.82	0.55–1.21	0.311
PREDIMED score	0.84	0.57–1.22	0.359
Current smoking	0.32	0.04–2.70	0.298
Hypertension	2.56	0.32–20.83	0.379
Diabetes	2.04	0.49–8.57	0.331
Dyslipidemia	1.83	0.37–9.13	0.463
Active or recent cancer (< 5 years)	5.56	1.07–28.92	**0.042**
Coronary artery disease	0.38	0.05–3.08	0.364
Prior stroke	0.67	0.08–5.46	0.707
sICAM-1 (× 10 ng/mL increase)	1.03	1.00–1.04	**0.002**
sVCAM-1 (× 10 ng/mL increase)	1.01	0.99–1.02	0.217
FKN (× 10 pg/mL increase)	1.33	1.05–1.62	**0.017**
*Multivariable analysis*			
	HR	95% CI	p
sICAM-1 (× 10 ng/mL increase)	1.03	1.00–1.05	**0.002**

*BMI* (body mass index); *PACE* (physician-based assessment and counseling for exercise); *sICAM-1* (soluble intercellular adhesion molecule-1); *sVCAM-1* (soluble vascular cell adhesion molecule-1); *FKN* (fractalkine). Backward stepwise multivariable Cox regression modeling was performed individually for each biomarker; *n* = 37; *p* < 0.05 indicates significant association. *BMI was divided into three categories (healthy weight, BMI 18.5 to < 25; overweight, BMI 25 to < 30; and obesity, BMI ≥ 30)

## Discussion

**Fig. 3:**
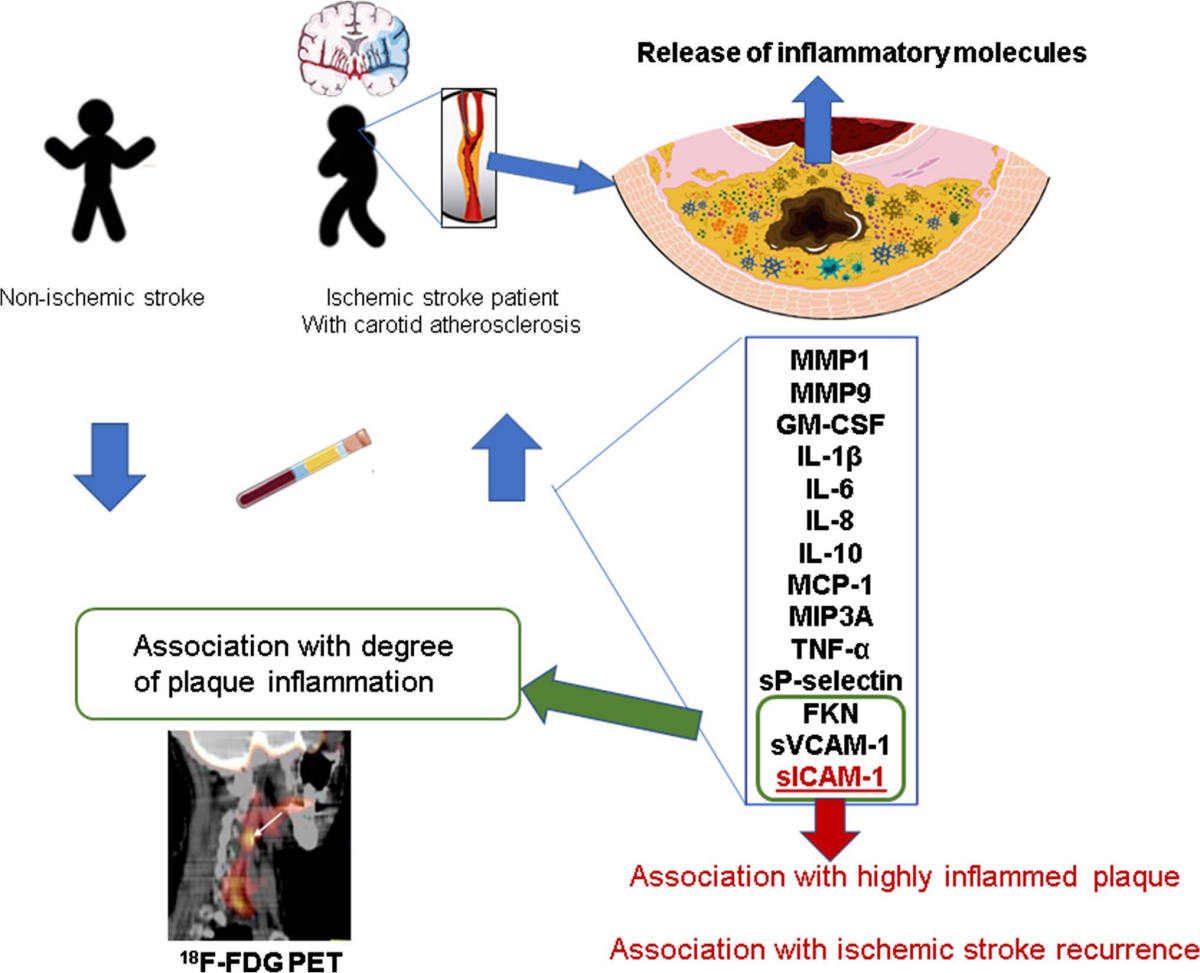
Main findings of the study. Several pro-inflammatory cytokines, chemokines, adhesion molecules, and metalloproteases were significantly elevated in plasma of the stroke patients compared to the healthy controls. These inflammatory mediators may be released into the circulation from inflamed atherosclerotic plaques, which become unstable and evolve to rupture. In agreement, among these molecules, FKN, sVCAM-1, and sICAM-1 concentrations were independently associated with the degree of plaque inflammation, evaluated by ^18^F-FDG PET. The plasma levels of sICAM-1 predicted with high sensibility the risk of finding highly inflamed carotid plaques and the recurrence of stroke within 1 year. Overall, these observations suggest that sICAM-1 is a bona fide marker for plaque inflammation and stroke recurrence

The present study provides new evidence of the potential role of certain inflammatory molecules in predicting carotid plaque inflammation and stroke recurrence in patients with a recent ischemic stroke and carotid atherosclerosis. The main findings are illustrated in Fig. 3, and they are as follows: (1) several pro-inflammatory cytokines, chemokines, adhesion molecules, and metalloproteases were significantly elevated in plasma of the stroke patients compared to the healthy controls; (2) FKN, sVCAM-1, and sICAM-1 concentrations were independently associated with the degree of plaque inflammation; and (3) the plasma levels of sICAM-1 predicted with high sensitivity the risk of finding highly inflamed carotid plaques and the recurrence of stroke within 1 year. Overall, these observations suggest that sICAM-1 is a bona fide marker for plaque inflammation and stroke recurrence.

We found that the levels of several pro-inflammatory cytokines and chemokines were increased in plasma from stroke patients presenting carotid atherosclerosis compared to the healthy controls. This is consistent with the fact that inflammation is a key factor throughout all stages of carotid atherosclerosis. It is noteworthy the increased concentration of plasma inflammatory markers is found in stroke patients at day 7, despite receiving antiplatelet agents and statins early after the index stroke according to the current guidelines on secondary stroke prevention. These drugs may have influenced the concentration of some of the inflammatory biomarkers analyzed in our study. For example, the elevation of some molecules, such as MMP2 and FABP4, may be more counteracted by drug therapy. We also found that the inflammatory molecules were elevated regardless of the stenosis degree. Therefore, their release seemed to depend more on the level of inflammation within the plaque than on the degree of carotid stenosis. Among these molecules, FKN, sVCAM-1, and sICAM-1 concentrations were independently associated with plaque inflammation, assessed by ^18^F-FDG uptake as SUVmax, a parameter that is a marker of carotid plaque inflammation in ischemic stroke patients [7]. On the other hand, in our study, brain injury does not seem a feasible source for inflammatory mediators, because according to the Rankin and the NIHSS scores (*md* = 2), the patients had minor strokes.

FKN, VCAM-1, and ICAM-1 are membrane proteins that can only be released to the circulation as soluble forms by activated cells in inflammation [19]. Cell-bound forms are mainly found on endothelial cells, although FKN is also expressed on neurons and smooth muscle cells and ICAM-1 on macrophages [19, 20], wherein they act as adhesion molecules. The role of adhesion molecules in the development of atherosclerosis through the recruiting of inflammatory cells to the lesions is well-described [21]. FKN expression has also been reported to induce macrophage survival in advanced atherosclerotic plaques [22]. In agreement, in a mouse model of atherosclerosis, the absence of FKN reduced the total lesion area and plaque macrophage content [23]. ICAM-1 was found to be highly expressed in atherosclerotic carotid plaques of symptomatic patients compared to asymptomatic plaques [24].

In inflammatory conditions, there is an increased expression of membrane-bound adhesion molecules and a release of their soluble forms [25]. However, information regarding the role of soluble adhesion molecules in atherosclerosis, particularly in the context of ischemic stroke, is scarce. In humans, the plasma concentration of sICAM-1 has been proposed as a marker of early atherosclerosis [26], and it has been related to the progression of atherosclerosis in mice [27]. Regarding ischemic stroke, some studies have reported increased plasma concentrations of sVCAM-1 and sICAM-1 in total ischemic stroke compared to healthy controls [28, 29], and a trend toward high plasma FKN levels was found in patients presenting moderate ischemic stroke (NIHSS < 3) [30], as was the case in the present study. However, until now, there has been a lack of information regarding a potential increase in these soluble molecules in the atherothrombotic subtype and their association with carotid plaque inflammation.

The shedding of FKN, sVCAM-1, and sICAM-1 from the membrane is an active process that is regulated by proteolytic enzyme activity, mainly MMPs [20, 31, 32]. As such, the increased concentration of soluble adhesion molecules found in the present study may have been partly caused by MMP activity, as indicated by the elevated plasma concentrations of MMP1 and MMP9 in our patients. In this context, plasma FKN, sVCAM-1, and sICAM-1 may mirror the presence of these inflammatory mediators within the plaque, where activated macrophages may play a key role by favoring their release into the circulation. Notably, we found an independent association between macrophage activation within the plaque, as assessed by ^18^F-FDG-PET, and the concentration of FKN, sVCAM-1, and especially sICAM-1, which was the only candidate molecule associated with highly inflamed carotid plaques (SUVmax ≥ 2.85 g/mL) independently of other confounding factors, such as BMI category. In this regard, sICAM-1 concentrations ≥ 240 ng/mL predicted with high sensitivity the presence of inflamed plaques according to ROC analysis, making sICAM-1 a much better predictor than CRP, which had a much lower area under the curve than sICAM-1.

We speculate that in patients with a recent ischemic stroke, sICAM-1 plasma concentrations may act as a surrogate marker of carotid plaque inflammation identifying patients who might be candidates for PET imaging. In addition, due to the protective effect of immunomodulation in atherosclerosis-related diseases [33], having a good surrogate marker of carotid inflammation might be very useful for monitoring patients’ responses to different treatments.

Interestingly, our study also showed a hitherto association between BMI category and SUVmax. This is in line with the fact that not only obese but also overweight patients have a low-grade chronic inflammatory state [34]. In this scenario, inflammation levels were associated with early carotid atherosclerosis in overweight patients, independently of insulin resistance [35]. Moreover, nonsmoking overweight subjects at an early age had an increased risk of death [36].

Since we found an association between plaque inflammation and FKN, sVCAM-1, and sICAM-1, we aimed to assess which of these molecules may also be associated with recurrence, because SUVmax had been associated with early stroke recurrence in patients with carotid stenosis [7]. Remarkably, sICAM-1, which independently associated with SUVmax ≥ 2.85 g/mL, was also associated with ischemic stroke recurrence within 1 year, independently of other confounding factors. However, there was no association when adjusting for SUVmax, which probably indicates that both biomarkers measured the same phenomenon. The association with stroke recurrence supports the idea that sICAM-1 measurement may be useful in secondary prevention by identifying patients at a high risk of recurrence.

sICAM-1 concentrations have been associated with an increased risk of total stroke in healthy women [37] and an increased risk of future coronary or ischemic stroke events in chronic coronary heart disease [38, 39]. In addition, sICAM-1 has also been associated with unfavorable outcome, evaluated as mRS, in ischemic patients [40, 41]. However, to our knowledge, the results of the present study show for the first time that the concentration of sICAM-1 is associated with stroke recurrence in patients with carotid stenosis. Of note, 40% of the patients in the ≥ 50% stenosis group underwent carotid revascularization, and thus, we cannot exclude that we underestimated the predictive power of sICAM-1 and the rest of candidate biomarkers. In other words, if revascularization had not occurred, this association would have likely been even greater.

As personalized medicine becomes more prevalent, new approaches are needed to help decision-making and provide the best treatment to stroke patients. Despite ^18^F-FDG PET is a well-validated diagnostic tool for identifying patients at high risk of stroke recurrence, some intrinsic limitations of this technique may hinder its implementation in the clinical practice such as its cost and the availability in some centers. In addition, although it reproducibly images plaque inflammation, ^18^F-FDG PET has limited spatial resolution and is unable to identify morphological plaque features (e.g., intraplaque hemorrhage or surface ulceration), which are associated with stroke recurrence. Within this context, our findings suggest that sICAM-1 measurement in ischemic stroke patients with carotid atherosclerosis may be useful for the following: (1) a cost-effective screening to determine which patients are candidates for ^18^F-FDG PET and (2) the evaluation of the efficacy of certain treatments or interventions, such as anti-inflammatory therapies. However, the present study has some limitations. First, the sample size for the study was small; therefore, the results should be corroborated using larger cohorts. Second, the results may not be replicated in severe strokes because the study population was biased toward minor strokes. And finally, we have compared concentrations of our candidate biomarkers with ^18^F-FDG uptake on PET/CT, and currently, more specific radiotracers exist for assessing inflammation.

## Conclusions

The findings of the present study indicate that, among the inflammatory molecules elevated after ischemic stroke and associated with carotid plaque inflammation, sICAM-1 is the best candidate biomarker for assessing plaque inflammation and predicting recurrence. Further investigations are warranted to more deeply understand the role of sICAM-1 and/or other related molecules and eventually incorporate those insights into the design of a diagnostic screening test to guide timely decision treatments in ischemic stroke patients. This knowledge would also be helpful in elucidating the pathophysiology of this complex and challenging disease, which would pave the way for the design of better therapeutic strategies.

## Supplementary Information

Below is the link to the electronic supplementary material.

Supplementary Table 1 (DOCX 24 KB)

Supplementary Table 2 (DOCX 25 KB)

Supplementary Table 3 (DOCX 18 KB)

## Data Availability

Additional data to that included in the manuscript can be provided under request.
