# Supplementary material for: Plasma sICAM-1 as a Biomarker of Carotid Plaque Inflammation in Patients with a Recent Ischemic Stroke
Source: Transl Stroke Res. 2022 Mar 2;13(5):745–56. doi: 10.1007/s12975-022-01002-x (PMC9391243; doi:10.1007/s12975-022-01002-x)
Supplement: Supplementary file 1 — Supplementary Table 1 (DOCX 24 KB) [file 12975_2022_1002_MOESM1_ESM.docx]

| Supplementary Table 1. Concentration of the inflammatory molecules according to the study population. | | | | | | |
| --- | --- | --- | --- | --- | --- | --- |
|  | <50% group (n=27) | ≥50% group (n=37) | *p* | All stroke patients (n=64) | Control group (n=27) | *p* |
| FABP4 (ng/mL),  md (IQR) | 108 (70-187) | 133 (54-253) | 0.533 | 123 (61-215) | 91 (48-180) | 0.430 |
| sP-Selectin (ng/mL), md (IQR) | 208 (136-326) | 183 (154-246) | 0.672 | 200 (139-281) | 70 (45-125) | **<0.001** |
| sICAM-1 (ng/mL), md (IQR) | 397 (224-705) | 309 (218-551) | 0.305 | 327 (218-578) | 213 (121-516) | **0.034** |
| sVCAM-1 (ng/mL), md (IQR) | 683 (556-819) | 693 (602-913) | 0.638 | 693 (565-899) | 494 (403-580) | **<0.001** |
| MMP1 (ng/mL),  md (IQR) | 3 (2-6) | 4 (2-4) | 0.744 | 3 (2-5) | 1 (1-1) | **<0.001** |
| MMP2 (ng/mL),  md (IQR) | 65 (60-81) | 61 (55-80) | 0.196 | 63 (57-80) | 60 (49-79) | 0.433 |
| MMP9 (ng/mL),  md (IQR) | 29 (19-56) | 42 (21-66) | 0.350 | 30 (20-59) | 21 (14-32) | **0.009** |
| FKN (pg/mL),  md (IQR) | 27 (20-40) | 26 (20-46) | 0.710 | 26 (20-43) | 17 (16-27) | **0.001** |
| GM-CSF (pg/mL), md (IQR) | 8 (7-11) | 8 (5-11) | 0.538 | 8 (6-11) | 6 (5-8) | **0.018** |
| IL-10 (pg/mL),  md (IQR) | 10 (7-16) | 10 (7-15) | 0.648 | 10 (7-16) | 6 (5-9) | **<0.001** |
| IL-1β (pg/mL),  md (IQR) | 0.9 (0.8-1.6) | 0.9 (0.5-1.5) | 0.709 | 0.9 (0.5-1.5) | 0.6 (0.4-0.8) | **0.002** |
| IL-6 (pg/mL),  md (IQR) | 0.6 (0.5-1.9) | 1.2 (0.4-2.9) | 0.195 | 0.9 (0.2-2.8) | 0.2 (0.1-1) | **0.010** |
| IL-8 (pg/mL),  md (IQR) | 2.4 (1.2-4.1) | 2.4 (1.3-5.7) | 0.665 | 2.4 (1.3-4.4) | 0.9 (0.3-1.5) | **<0.001** |
| MIP3A (pg/mL),  md (IQR) | 10 (7-14) | 8 (6-13) | 0.236 | 8 (6-13) | 4 (2-9) | **<0.001** |
| TNF-α (pg/mL),  md (IQR) | 5 (3-7) | 4 (3-6) | 0.681 | 4 (3-6) | 3 (2-3) | **<0.001** |
| MCP-1 (pg/mL),  md (IQR) | 112 (72-134) | 118 (87-159) | 0.379 | 115 (84-155) | 87 (62-107) | **0.013** |

FABP4 (fatty acid binding protein 4); sP-selectin (soluble P-selectin); sICAM-1 (soluble intercellular adhesion molecule-1); sVCAM-1 (soluble vascular cell adhesion molecule-1); MMP (matrix metalloproteinases); FKN (fractalkine); GM-CSF (granulocyte-macrophage colony-stimulating factor); IL (interleukin); MIP3A (macrophage inflammatory protein-3); TNF-α (tumor necrosis factor-α); MCP-1 (monocyte chemoattractant protein-1).

Wilcoxon rank-sum test was used to compare between groups; *p*<0.05 indicates significant differences.
