# Supplementary material for: Plasma sICAM-1 as a Biomarker of Carotid Plaque Inflammation in Patients with a Recent Ischemic Stroke
Source: Transl Stroke Res. 2022 Mar 2;13(5):745–56. doi: 10.1007/s12975-022-01002-x (PMC9391243; doi:10.1007/s12975-022-01002-x)
Supplement: Supplementary file 2 — Supplementary Table 2 (DOCX 25 KB) [file 12975_2022_1002_MOESM2_ESM.docx]

| Supplementary Table 2. Bivariate and multivariable logistic regression analyses of predictors of carotid plaque SUVmax ≥2.85 g/L | | | |
| --- | --- | --- | --- |
| *Bivariate analysis* | | | |
|  | OR | 95% CI | *p* |
| Age | 1.05 | 0.98-1.13 | 0.133 |
| Sex (female) | 1.61 | 0.44-5.96 | 0.475 |
| BMI category* | 2.79 | 1.07-7.27 | **0.036** |
| PACE score | 0.60 | 0.39-0.92 | **0.019** |
| PREDIMED score | 0.89 | 0.68-1.16 | 0.375 |
| Current smoking | 0.15 | 0.02-1.32 | 0.088 |
| Diabetes | 2.57 | 0.76-8.76 | 0.131 |
| Dyslipidemia | 0.81 | 0.23-2.94 | 0.754 |
| Active or recent cancer (<5years) | 9.25 | 0.88-97.47 | 0.064 |
| Coronary artery disease | 0.61 | 0.14-2.60 | 0.508 |
| Prior stroke | 0.27 | 0.03-2.35 | 0.235 |
| Prior antiplatelet therapy | 0.75 | 0.22-2.49 | 0.633 |
| Prior statin therapy | 1.09 | 0.32-3.69 | 0.889 |
| Carotid stenosis ≥50% | 3.06 | 0.82-11.32 | 0.095 |
| Triglycerides (mg/dL) | 1.01 | 0.99-1.02 | 0.432 |
| Total cholesterol | 1.00 | 0.99-1.02 | 0.730 |
| LDLc (mg/dL) | 0.99 | 0.98-1.02 | 0.936 |
| HDLc (mg/dL) | 1.01 | 0.97-1.05 | 0.697 |
| sICAM-1 (ng/mL) | 1.00 | 0.99-1.00 | 0.078 |
| sVCAM-1 (ng/mL) | 1.00 | 0.99-1.00 | 0.185 |
| FKN (pg/mL) | 1.03 | 1.00-1.07 | **0.044** |
| *Multivariable analysis* | | | |
| *Model 1 (including sICAM-1)* | OR | 95% CI | *p* |
| sICAM-1 (x10 ng/mL increase) | 1.02 | 1.00-1.03 | **0.020** |
| PACE score | 0.60 | 0.36-0.98 | **0.043** |
| BMI category (x1 category increase)* | 3.56 | 1.02-12.3 | **0.045** |
| Carotid stenosis ≥50% | 6.07 | 0.88-41.90 | 0.067 |
| *Model 2 (including FKN) †* |  |  |  |
| PACE score | 0.60 | 0.38-0.94 | **0.027** |
| BMI category (x1 category increase) | 2.53 | 0.92-6.98 | 0.072 |

BMI (body mass index); PACE (physician-based assessment and counseling for exercise); LDLc (low-density lipoprotein cholesterol); HDLc (high-density lipoprotein cholesterol); sICAM-1 (soluble intercellular adhesion molecule-1); sVCAM-1 (soluble vascular cell adhesion molecule-1); FKN (fractalkine).

Backward stepwise multivariable logistic regression modeling was performed individually for each biomarker; n=53; *p*<0.05 indicates significant association.

* BMI was divided into three categories (healthy weight: BMI 18.5 to <25, overweight: BMI 25 to <30, and obesity: BMI ≥30)

†Fractalkine is removed from the final model with a p=0.133
