# Supplementary material for: Plasma sICAM-1 as a Biomarker of Carotid Plaque Inflammation in Patients with a Recent Ischemic Stroke
Source: Transl Stroke Res. 2022 Mar 2;13(5):745–56. doi: 10.1007/s12975-022-01002-x (PMC9391243; doi:10.1007/s12975-022-01002-x)
Supplement: Supplementary file 3 — Supplementary Table 3 (DOCX 18 KB) [file 12975_2022_1002_MOESM3_ESM.docx]

| Supplementary Table 3. Association between plasmatic concentrations of sICAM-1, sVCAM-1 and FKN and carotid plaque risk features by ultrasonography | | | | | | | | | |
| --- | --- | --- | --- | --- | --- | --- | --- | --- | --- |
|  | Plaque echolucency* | |  | Plaque surface | |  | Plaque stenosis** | | |
|  | Hypoechoic (n=20) | Hyperechoic (n=40) | p | Irregular (n=39) | Regular (n=21) | p | ≥50% (n=37) | <50% (n=22) | p |
| sICAM-1 (ng/mL), md (IQR) | 400  (213-670) | 326  (166-551) | 0.462 | 395  (220-662) | 299  (205-569) | 0.781 | 309  (218-551) | 397  (224-705) | 0.305 |
| sVCAM-1 (ng/mL), md (IQR) | 628  (506-725) | 743  (597-913) | 0.130 | 649  (597-833) | 720  (556-899) | 0.914 | 693  (602-913) | 683  (556-819) | 0.638 |
| Fraktalkine (pg/mL), md (IQR) | 43  (23-53) | 24  (18-38) | **0.025** | 31  (20-45) | 22  (18-42) | 0.599 | 26  (20-46) | 27  (20-40) | 0.710 |
| SUVmax (g/mL), md (IQR) | 3.14  (2.41-3.46) | 2.45  (2.27-2.74) | **0.009** | 2.74  (2.40-3.44) | 2.41  (2.26-2.71) | **0.047** | 2.74  (2.41-3.23) | 2.39 (2.23-2.69) | **0.034** |

IQR=Interquartile Range; FKN=Fraktalkine; md= Median; sICAM-1=Soluble Intercellular Adhesion Molecule-1; SUVmax=Maximal Standardized Uptake Value; sVCAM-1=Soluble Vascular Adhesion Molecule-1.

*A plaque was classified as hypoechoic if >50% of its content showed lower echogenicity than the muscle (sternocleidomastoid).

**Plaque stenosis was graded according to hemodynamic criteria [14]
